# Imagery or meaning? Evidence for a semantic origin of category-specific brain activity in metabolic imaging

**DOI:** 10.1111/j.1460-9568.2008.06143.x

**Published:** 2008-04-01

**Authors:** Olaf Hauk, Matthew H Davis, Ferath Kherif, Friedemann Pulvermüller

**Affiliations:** 1 Medical Research Council, Cognition and Brain Sciences Unit Cambridge, UK; 2 University College London, Wellcome Trust Functional Imaging Laboratory London, UK

**Keywords:** action-relatedness, cell assemblies, imageability, multiple regression, visual word recognition, word frequency

## Abstract

Category-specific brain activation distinguishing between semantic word types has imposed challenges on theories of semantic representations and processes. However, existing metabolic imaging data are still ambiguous about whether these category-specific activations reflect processes involved in accessing the semantic representation of the stimuli, or secondary processes such as deliberate mental imagery. Further information about the response characteristics of category-specific activation is still required. Our study for the first time investigated the differential impact of word frequency on functional magnetic resonance imaging (fMRI) responses to action-related words and visually related words, respectively. First, we corroborated previous results showing that action-relatedness modulates neural responses in action-related areas, while word imageability modulates activation in object processing areas. Second, we provide novel results showing that activation negatively correlated with word frequency in the left fusiform gyrus was specific for visually related words, while in the left middle temporal gyrus word frequency effects emerged only for action-related words. Following the dominant view in the literature that effects of word frequency mainly reflect access to lexico-semantic information, we suggest that category-specific brain activation reflects distributed neuronal ensembles, which ground language and concepts in perception-action systems of the human brain. Our approach can be applied to any event-related data using single-stimulus presentation, and allows a detailed characterization of the functional role of category-specific activation patterns.

## Introduction

Are mental representations of word meaning abstract, symbolic entities, or do perceptual and motoric representations play a critical role in ‘grounding’ word meaning in representations of the external objects and events that words denote? This question has a long history in psychology (Harnad, 1990; Paivio, 1991; Barsalou, 1999; Lakoff & Johnson, 1999; Pulvermüller, 1999; Glenberg & Kaschak, 2002; Pylyshyn, 2002; Rogers *et al.*, 2004), and numerous neuroscientific studies have sought an answer by localizing the neuronal networks underlying semantic representations (see, e.g. Barsalou *et al*., 2003; Caramazza & Mahon, 2003; Pulvermüller, 2005; Martin, 2007). Several studies have shown that activation evoked in tasks that involve access to semantic knowledge can include motor and sensory processing areas (Martin *et al*., 1996; Kiefer & Spitzer, 2001; Noppeney & Price, 2002; Hauk *et al*., 2004; Tettamanti *et al.*, 2005; Pulvermüller & Hauk, 2006). However, measurement of the slow haemodynamic response provides low temporal resolution and therefore previous neuroimaging results have not been able to distinguish between activation evoked by elementary recognition processes (accessing the meaning of the word ‘hammer’, for example) and ‘epiphenomenal’ post-recognition processes, such as deliberate imagery (e.g. imagining using a hammer) or ‘post-understanding translation’ (Glenberg & Kaschak, 2002).

The main goal of this study was to gain new insights into the response characteristics of word category-specific brain activation, which could help distinguish between theories in which: (1) category-specific activation is critical for recognition; or (2) accounts in which such specific activation arises from post-recognition processes such as imagery. We assessed the effect of a lexical variable, the frequency of occurrence of written words, on neural responses to words in different categories, namely visually and action-related words (e.g. ‘sun’ and ‘kick’, respectively). Effects of word frequency on lexical decision times have been reported for several decades (e.g. Whaley, 1978; Gernsbacher, 1984). Although there has been some debate about the exact locus of the word frequency effect (e.g. Balota & Chumbley, 1984; Mccann *et al.*, 1988; Paap & Johansen, 1994), recent behavioural studies have provided strong evidence that early word recognition processes are sensitive to word frequency (Allen *et al*., 2005; Cleland *et al*., 2006). This is further confirmed by a number of electrophysiological studies that reported word frequency effects within 200 ms after stimulus onset (Sereno *et al*., 1998; Assadollahi & Pulvermüller, 2003; Hauk & Pulvermüller, 2004a; Dambacher *et al*., 2006; Hauk *et al*., 2006). Importantly for our study, word frequency is not usually considered as a crucial factor in mental imagery or simulation (Paivio, 1986; Pylyshyn, 2002), consistent with the intuition that synonyms that differ in frequency (e.g. ‘baby’, ‘infant’) should not differ with respect to the effort it takes to evoke a mental image or to recall previous episodic experiences once word identification is complete.

In accordance with the negative correlation generally observed between word frequency and behavioural response times, electrophysiological (Sereno *et al*., 1998; Assadollahi & Pulvermüller, 2003; Hauk & Pulvermüller, 2004a; Dambacher *et al*., 2006; Hauk *et al*., 2006) as well as metabolic (Fiebach *et al*., 2002; Chee *et al*., 2003; Kuo *et al.*, 2003; Kronbichler *et al.*, 2004; Carreiras *et al*., 2006) neuroimaging research has consistently shown that rare words elicit stronger brain responses than words with high word frequency. On the basis of semantic accounts of category specificity, we therefore hypothesized that word frequency effects should correlate negatively with brain activation in object-related areas (e.g. in fusiform gyrus) for visually related words, and in action-related areas (such as middle temporal and precentral gyrus) for action-related words. Our approach allows more precise conclusions about the functional role of category-specific activation patterns than has been available from metabolic imaging techniques so far.

## Materials and methods

### Stimuli and experimental design

Twenty one right-handed native speakers of English participated in the study (10 female and 11 male; mean age ± SD = 24.5 ± 5.3 years). They had no history of neurological or psychiatric illness or drug abuse. Ethical approval was obtained from the Cambridge Local Research Ethics Committee. This task has been used successfully in several previous neuroimaging studies on visual word recognition (Mechelli *et al*., 2003; Hauk *et al*., 2004; Kronbichler *et al.*, 2004). Stimuli were flashed briefly on the screen for 100 ms in order to minimize eye movements and variance in stimulus processing times. The stimulus onset asynchrony (SOA) was 2.5 s. Two-hundred and fifty monosyllabic and mono-morphemic English word stimuli were employed in the study. One-hundred and fifty referred to bodily actions (e.g. ‘grasp’, ‘limp’, ‘bite’), and 100 to objects and visual attributes (e.g. ‘snow’, ‘blond’, ‘cube’). These categories were matched on average familiarity, number of letters and number of phonemes. One-hundred and fifty baseline trials consisting of strings of hash marks varying in length were interspersed among the word stimuli. The average length of words and hash marks was matched. In addition, 50 null events were included in which a fixation cross remained on the screen. Two pseudo-randomized stimulus sequences were alternated between subjects.

For each of the 250 word stimuli, we obtained 21 psycholinguistic parameters, either from the CELEX database (such as word form, lemma, bi- and trigram frequencies; Baayen *et al*., 1993) or from a separate rating study (such as action-relatedness, imageability and familiarity; Hauk & Pulvermüller, 2004b). Some of these variables are highly correlated with each other (such as word form frequency, lemma frequency and familiarity), and their effects can therefore be impossible to estimate independently from each other. Furthermore, the questions underlying our study do not require the estimation of effects for each of these variables individually.

In order to reduce the information available for our stimulus set to a tractable number of variables for the multiple regression analysis, we combined some of the parameters into groups based on their functional relatedness and their intercorrelation pattern (see below). For each of these groups, every variable was z-normalized across stimuli, and a principal component analysis was computed. The first principal component of each group entered the multiple regression analysis. The final variables were: (1) Length and Neighbourhood Size; created from the parameters number of letters and neighbourhood size, which were strongly negatively correlated; (2) Typicality; created from orthographic bigram and trigram frequency (strongly positively correlated); (3) Frequency; created from word form frequency, lemma frequency and familiarity (strongly positively correlated); (4) Action-relatedness; created from body- and action-relatedness (strongly positively correlated); (5) Imageability; created from imageability, concreteness and visually relatedness (strongly positively correlated). In the following we will use capital initial letters if we explicitly refer to those variables that entered our analysis (e.g. ‘Frequency’), but use small initial letters if we refer to the variable in general (such as word ‘frequency’). Note also the different use of the terms word ‘variables’ and ‘categories’: the former refers to the constituents of the regression analysis (e.g. Frequency, Action-relatedness), the latter to the different word groups in the factorial analysis (action-related and visually related words).

### Data acquisition and analysis

Twenty-one monolingual, right-handed, healthy native English speakers participated in the study. Their mean age was 24.5 years (SD 5.3), and their handedness score (from a reduced version of the Oldfield handedness inventory; Oldfield, 1971) was 87 (SD 15). Scanning took place in a 3T Bruker MR system using a head coil. Echo planar images (EPI) were acquired using a TR = 3.02 s, TE = 27 ms and a flip angle of 90 degrees. Reconstructed images consisted of 21 slices covering the whole brain, with slice thickness 4 mm, interslice distance 1 mm, field-of-view 25 cm and in-plane resolution 128*128. Seven subjects had participated in a similar electroencephalogram (EEG) experiment before the functional magnetic resonance imaging (fMRI) session (average delay 18 days, SD 11 days). The remaining 14 data sets were also part of the study of Hauk *et al*. (2004). Images were corrected for slice timing, and then realigned to the first image using sinc interpolation. Phasemaps were used to correct for inaccuracies resulting from inhomogeneities in the magnetic field (Jezzard & Balaban, 1995; Cusack *et al*., 2003). Any non-brain parts were removed from the T1-weighted structural images using a surface model approach (‘skull-stripping’; Smith, 2002). The EPI images were coregistered to these skull-stripped structural T1-images using a mutual information coregistration procedure (Maes *et al*., 1997). The structural MRI was normalized to the 152-subject T1 template of the Montreal Neurological Institute (MNI). The resulting transformation parameters were applied to the coregistered EPI images. Images were resampled with a spatial resolution of 2 × 2 × 2 mm^3^, and spatially smoothed with a 12-mm full-width half-maximum Gaussian kernel. This was done to capture variability across subjects, but to be able to separate activation in brain areas that are typically several centimeters apart. After global normalization of data from separate sessions, single-subject statistical contrasts were computed using a parametric general linear model (Buchel *et al*., 1998; Friston *et al.*, 1998). Low-frequency noise was removed with a high-pass filter (time constant 60 s). Imaging data were processed using SPM99 software (Wellcome Department of Cognitive Neurology, London, UK).

### Multiple regression analysis

One important methodological concern in many previous neuroimaging studies on word recognition is that the statistical analysis methods employed assess the consistency of activation differences between two classes of words over a population of subjects, but do not assess the consistency (or otherwise) of activation differences over a population of linguistic items. It has been argued previously that multiple linear regression designs in combination with random effects group statistics optimally account for appropriate sources of between-subject variance (Lorch & Myers, 1990). We therefore applied two linear regression designs to our data, each of which was optimized for a specific purpose.

**Fig. 1:**
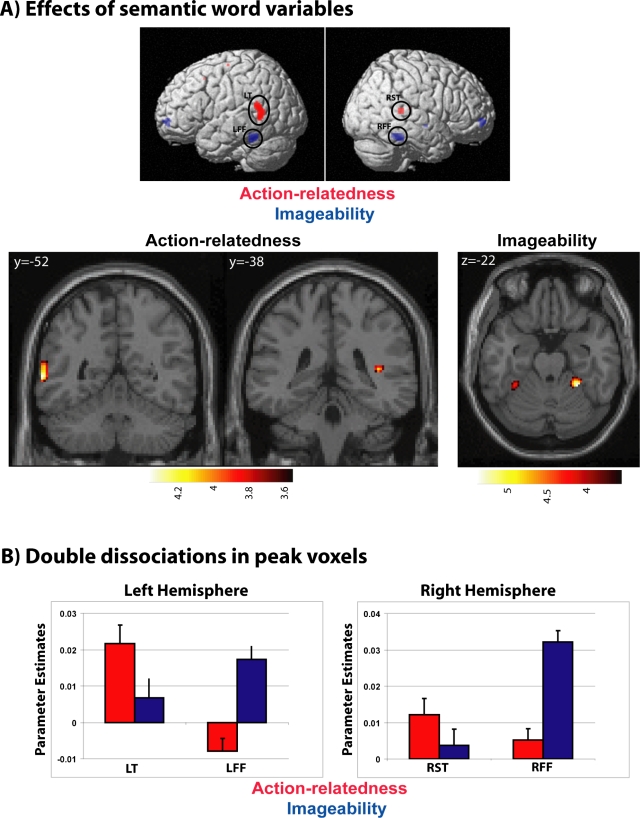
Brain activation modulated by semantic variables. (A) Activation peaks for the variables Action-relatedness (red) as well as Imageability (blue), projected on the surface (top) and on cortical slices (bottom) of a standard brain. Encircled activation spots were significant after SVC, with volumes of interest defined based on previous publications. Activation is displayed at a statistical threshold *P* < 0.001 uncorrected. Colour coding reflects *t*-values. Coordinates and statistics are provided in Table 1. (B) Parameter estimates (arbitrary units) for the two semantic variables in peak voxels of left temporal gyrus (LT) and left fusiform gyrus (LFF), as well as right superior temporal gyrus (RST) and right fusiform gyrus (RFF). The error bars represent within-subject standard errors.

In a first analysis, we sought to corroborate previous findings on action-relatedness and imageability. The five word variables described above were used as simultaneous linear regressors across all words, i.e. effects of variables that were not of interest for this study (length and orthographic typicality) were partialled out. Note that in order to detect effects of Action-relatedness and Imageability, the stimuli must exhibit sufficient variability with respect to these variables. This analysis therefore applied regression to all words including visually related and action-related words, as each of them by itself was chosen to be relatively homogenous with respect to one of the two variables. This yielded the results presented in Fig. 1 and Table 1.

**Table 1:** MNI coordinates and Z-scores for voxels that showed significant activation for all words compared with a low level baseline (hash marks), and positive correlations with Imageability or Action-relatedness, respectively

Region	x	y	z	*Z*-score
All Words > Hash Marks				
L fusiform	−42	−46	−22	5.22
L mid temporal	−56	−50	−4	3.24
L precentral	−54	12	32	4.23
L inf frontal, tri	−44	24	16	4.19
L inf frontal, tri	−54	24	0	3.85
L postcentral	−36	−24	50	3.63
L inf frontal, orb	−32	30	−14	3.20
L SMA	−2	18	48	3.14
L inf parietal	−46	−42	56	3.12
Imageability				
L fusiform*	−34	−44	−22	3.57
R fusiform*	28	−44	−22	4.18
Thalamus	0	−22	6	3.43
Thalamus	2	−12	2	3.19
R med frontal, orb	4	58	−4	3.55
Action-relatedness				
L mid temporal*	−62	−52	4	3.61
L sup temporal*	−62	−48	16	3.54
L precentral*	−26	−14	64	3.11
R sup temporal*	40	−38	10	3.47
Thalamus	4	−14	6	3.27
Thalamus	16	−28	12	3.18
L SMA	−12	14	48	3.13

* Areas which fell in volumes of interest defined on the basis of previous publications. g, gyrus; L, left; med, medial; mid, middle; oper, operculum; orb, orbital; R, right; sup, superior; temp, temporal; tri, triangularis.

**Fig. 2:**
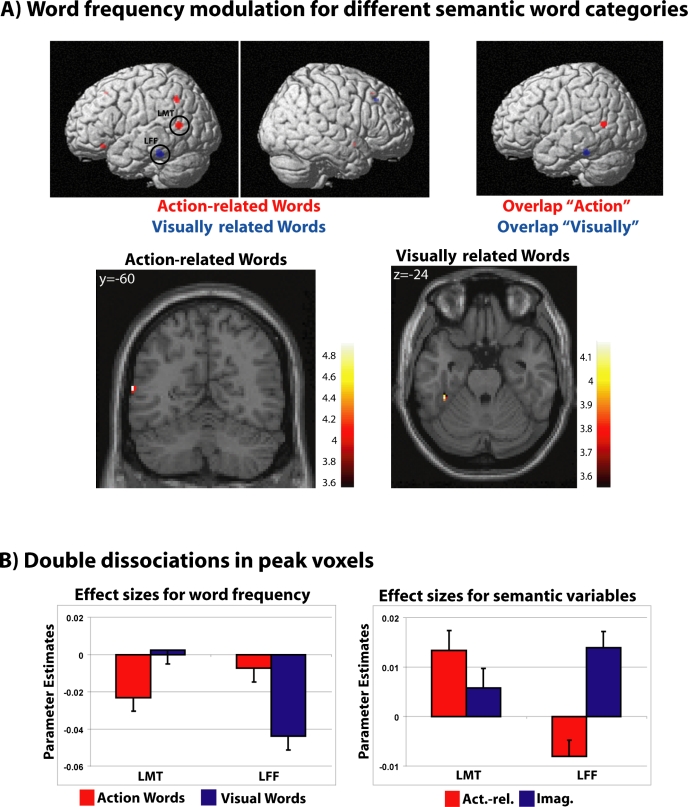
Category-specific effects of word frequency. (A) Brain areas significantly negatively correlated with Frequency for action-related words (red) and visually related words (blue) separately, projected on the surface (top) and on cortical slices (bottom) of a standard brain. Activation is displayed at a statistical threshold *P* < 0.001 uncorrected. The rendered image at the upper right represents the overlap of activations from this analysis with that presented in Fig. 1A at threshold *P* < 0.01. Colour coding reflects *t*-values. Coordinates and statistics are provided in Table 2. (B) The left diagram presents parameter estimates for the variable Frequency for action-related words and visually related words separately, for peak voxels in the left middle temporal gyrus (LMT) and left fusiform gyrus (LFF). The right diagram shows parameter estimates for the same voxels but for the variables Action-relatedness (Act.-rel.) and Imageability (Imag.) across all words independent of word category. The error bars represent within-subject standard errors.

The crucial prediction of our study was tested in a separate analysis that looked at the two critical word categories separately. Words were grouped into action- and visually related words, which were specified as different columns of the design matrix. Each of these two word groups was assigned the same five simultaneous regressors as in the first analysis, including the crucial variable, Frequency. This model yielded the results presented in Fig. 2 and Table 2.

**Table 2:** MNI coordinates and *Z*-scores for peak voxels that showed significant negative correlation with Frequency, computed for action-related and visually related words separately, as well as for all words

Region	x	y	z	*Z*-score
Frequency − action-related words only				
L mid temporal*	−60	−60	10	3.94
L angular	−46	−56	40	3.52
L inf frontal, orb	−28	28	−14	3.36
R SMA	8	24	48	3.23
Frequency − visually related words only				
L fusiform*	−38	−38	−24	3.49
R sup frontal	18	30	40	3.21
Frequency − all words				
L fusiform*	−36	−32	−18	5.27
	−28	−36	−22	4.54
	−18	−36	−26	4.29
L inf frontal, orb*	−30	28	−12	4.15
	−20	26	4	3.89
L Insula*	−32	24	8	3.66
L inf frontal, oper*	−38	14	30	3.65
R inf frontal, orb*	32	28	−6	3.75
R Insula*	30	26	10	3.46

Symbols and abbreviations as for Table 1.

Group data were analysed with a random-effects analysis. We will focus our main interpretations on activation peaks that both reached an uncorrected significance level of *P* < 0.001 (used for display), and were significant at *P* < 0.05 corrected after small volume correction (SVC) for volumes of interest that were defined based on previous findings (see below). Stereotaxic coordinates for voxels with maximal *z*-values within activation clusters are reported in the coordinate system of the standard brain of the MNI. Anatomical labels of nearest cortical grey matter for peak coordinates were obtained from the MRIcron software (http://www.sph.sc.edu/comd/rorden/mricro.html), based on the anatomical parcellation of the MNI brain published by Tzourio-Mazoyer *et al.* (2002). To address questions about the specificity of activations across several activation clusters, we computed parameter estimates for peak voxels for each individual subject using standard procedures implemented in the MarsBar software (Brett *et al*., 2002), and subjected them to anovas including a factor representing spatial location of the voxels. Error bars in the corresponding graphs indicate within-subject standard errors for the comparison of two conditions at each location, i.e. between-subject variability has been removed before computing the standard error. This is appropriate for displaying confidence intervals in repeated-measures designs (Loftus & Masson, 1994).

### SVC

We formulated several hypotheses about activation patterns associated with our word variables. These were tested using SVC for spheres with radius 20 mm centred at coordinates taken from the literature, as will be described below. Where necessary, mean coordinates were computed in the coordinate system reported in the original study, and transformed to MNI coordinates afterwards. The hypotheses for these volumes were independent of each other, and were therefore tested separately.

We predicted that Imageability would activate object processing areas in the fusiform gyrus. We therefore compared our activation patterns to those of a study that reported activation for different kinds of objects, namely animals and tools, in the bilateral fusiform gyrus (Chao *et al*., 1999). The mean coordinates for object-related activation in ventral and lateral fusiform gyrus in this study were 33/−54/−16 (RH) and −33/−54/−16 (LH).

Action-relatedness should activate motor areas in the middle temporal gyrus and frontal cortex. The middle temporal cortex has been found to be activated by action-related objects and words in several previous studies (Martin *et al*., 1996; Chao *et al*., 1999; Grezes *et al*., 2003; Davis *et al*., 2004). For consistency reasons, we chose coordinates from the same study as above that were activated more strongly for tools than for animals, i.e. −47/−55/3 (LH) and 45/−54/3 (RH) (Chao *et al*., 1999). As a frontal motor region, we chose the coordinates for activations evoked by finger movements reported by Hauk *et al*. (2004), which were −36/−8/60 (LH) and 38/−20/48 (RH). Given that the present study employed a subset of subjects who also performed the motor localizer task published by Hauk *et al*. (2004), the corresponding coordinates are more likely to reflect brain areas involved in hand-action processing for our subjects than values from the literature.

### Double dissociations between locations and word categories

The mass-univariate statistical approach applied to visually related and action-related words separately in the above SPM analysis cannot fully determine whether the apparent differences between word categories are due to the selected statistical threshold, or reflect a true double dissociation of the factors Location (fusiform vs middle temporal) and semantic variable (Imageability vs Action-relatedness). A further question is whether the Frequency modulations obtained by the second analysis indeed overlap with the activations from the first analysis.

Comparisons of effect sizes between different brain regions are complicated by the fact that the blood-oxygen-level-dependent (BOLD) response may vary due to differences in vasculature, neuron-vasculature coupling, and other anatomical or physiological factors unrelated to the experimental manipulations. However, such factors would not be able to explain a cross-over interaction or double dissociation for the factors location and word category, which are the crucial predictions of our study (see Henson, 2006 for a discussion of possible inferences that can be drawn from functional imaging data). We therefore performed the following tests:

1. Double dissociation for semantic variables. We tested for a double dissociation between different brain loci and semantic variables (Imageability, Action-relatedness). For this purpose, we performed a 2-by-2 anova with the factors Location (mean value for two peak voxels in left middle/superior temporal gyrus, as well as fusiform gyrus, from the first analysis) and Semantic Variable (Action-relatedness and Imageability).
2. Double dissociation of Frequency modulation. We tested for a double dissociation between Frequency modulation at different brain loci and word categories (visually and action-related words). For this purpose, we performed a 2-by-2 anova with the factors Location (peak voxel in left middle temporal gyrus, as well as fusiform gyrus) and Word Category (Frequency modulation for visually related vs action-related words).
3. Overlap of effects for semantic variables and Frequency. In order to test for an activation overlap, we extracted the parameter estimates for the variables Action-relatedness and Imageability in the voxels exhibiting the strongest modulation by Frequency in the second analysis. These values were entered into a 2-by-2 anova with the factors Location (fusiform vs middle temporal gyrus) and Semantic Variable.

## Results

### Analysis of all words

We contrasted activation for all words to the baseline condition. The most dominant activation peaks occurred in the left hemisphere, i.e. in the left fusiform, precentral, middle temporal and inferior frontal gyrus (Table 1). Table 2 includes the coordinates of the most dominant peak voxels that showed negative correlation with Frequency across all words (i.e. action-related and visually related words combined). The main areas modulated by Frequency were located in the left and right inferior frontal cortex and left fusiform cortex. Splitting the whole stimulus set into subcategories (action- and visually related words in our case) is meant to increase sensitivity for detecting effects that are specific to these categories, but also means reducing statistical power for the analysis of effects that are characteristic for the whole stimulus set (all words). Therefore, this study focused primarily on category-specific effects of word frequency. The categories targeted here are action- and visually related words; a more detailed discussion of the results for the all word analysis will be presented elsewhere.

### Effects of semantic variables

The most significant correlation between Imageability and brain activation occurred in the left fusiform gyrus, and in an almost symmetrical right hemispheric area (Fig. 1 and Table 1). Both of these peaks fell in the vicinity (∼10 mm) of the mean coordinates computed from Chao *et al*. (1999). A *t*-test on parameter estimates for these peak voxels in the left and right hemisphere did not reveal a significant difference (*t*_20_ = −1.5, *P* > 0.1).

Action-relatedness correlated positively with brain activation in the left middle temporal gyrus and left superior temporal gyrus, near the corresponding action-related areas reported by Chao *et al*. (1999). Interestingly, the peak in the left middle temporal gyrus was approximately 10 mm away from the location at which Davis *et al*. (2004) reported positive correlation with action-relatedness. SVC analysis around a symmetrical location in the right temporal cortex revealed a marginally significant activation near the right superior temporal gyrus. A *t*-test comparing parameter estimates between these peak voxels in the left and right hemisphere for Action-relatedness did not reveal a significant difference (*t*_20_ = 0.77, *P* > 0.4).

Further activation for Action-relatedness was found in a premotor area of the left middle frontal gyrus, i.e. an area corresponding to the dominant hand. It was located approximately 12 mm from the left dorsolateral activation spot related to finger movements reported in Hauk *et al*. (2004). However, although this activation was significant at an uncorrected threshold of *P* < 0.001, it was only marginally significant in the SVC analysis.

### Correlations with Frequency for semantic word categories

In a second analysis step, a multiple regression with separate columns encoding action-related and visually related words was conducted. The peak voxels that showed significant negative correlations with Frequency are listed in Table 2, and the activation peaks rendered on a standard brain surface are shown in Fig. 2. For action words, the most dominant voxel occurred in the left middle temporal gyrus, 10 mm from the peak voxel in the left middle temporal gyrus that correlated significantly with Action-relatedness. Further voxels showing a significant negative correlation with Frequency for action words were found in the left inferior parietal lobe, left inferior and right medial frontal gyrus. The corresponding analysis for visually related words yielded two significant activation spots. The dominant one occurred in the left fusiform gyrus, 8 mm from the Imageability peak voxel in the left fusiform gyrus described above. Another area that showed significant negative correlation with Frequency for visually related words was located in the right middle frontal gyrus.

A double dissociation for semantic variables was substantiated by a 2-by-2 anova with the factors Location and Semantic Variable, which revealed a significant interaction (*F*_1,20_ = 9.67, *P* < 0.01). Action-relatedness did not differ significantly from zero in the peak voxel for Imageability in the left fusiform gyrus, and analogously for Imageability in the left middle/superior temporal gyrus (both *t*-tests yielded *P* > 0.2). The interaction for right-hemispheric voxels (right superior temporal and right fusiform gyrus) yielded a qualitatively similar result (*F*_1,20_ = 11.95, *P* < 0.01). The corresponding parameter estimates are presented in Fig. 1. Accordingly, a double dissociation for Frequency modulation was documented by a 2-by-2 anova on parameter estimates, including the factors Location (peaks with largest Frequency modulation) and Word Category (action- and visually related), which yielded a significant interaction (*F*_1,20_ = 8.15, *P* < 0.02). Overlap of effects for semantic variables and Frequency was revealed by a 2-by-2 anova with the factors Semantic Variable (Action-relatedness and Imageability) and Location (peak voxels of modulation with Frequency, for action-related and visually related words, respectively), resulting in a significant interaction (*F*_1,20_ = 6.71, *P* < 0.02). Action-relatedness significantly differed from zero only in the left middle temporal gyrus, while the same was true for Imageability only in the left fusiform gyrus (all *P* < 0.05 or *P* > 0.1, respectively; Fig. 2). This overlap is further illustrated in Fig. 2A.

## Discussion

We studied differences in brain activation for words with different semantic associations in an event-related fMRI study using a silent reading task. The main novel finding consists of a differential modulation of activation for visually and action-related words by the frequency of the words' occurrence, a variable commonly associated with word identification and lexico-semantic access. We also corroborated previous findings of category-specific brain activation in word recognition with respect to imageability and action-relatedness in the fusiform and lateral temporal lobe as well as premotor cortex, respectively. The negative correlation observed between Frequency and neural activity for written words was both specific to either visually or action-related words, and localized in brain areas that also showed differential effects for Imageability and Action-relatedness. For action words, the maximum negative correlation with Frequency fell within about 10 mm of the peak activations for Action-relatedness in the left middle temporal gyrus. Correspondingly, for visually related words and Imageability these activations were about 10 mm apart in the left fusiform gyrus. Furthermore, we found an overlap of effects for category-specific Frequency modulations on the one hand, and Action-relatedness and Imageability on the other.

### Effects of word frequency

A number of previous metabolic neuroimaging studies have investigated effects of word frequency on brain activation (see Fiebach *et al*., 2002; Chee *et al*., 2003; Kuo *et al.*, 2003; Kronbichler *et al.*, 2004; Carreiras *et al*., 2006 for recent examples). They reported effects of word frequency mainly for ‘classical’ language-related areas, such as in the left inferior frontal or left inferior temporal cortex, but not for areas related to action- or object-related processing. However, these studies did not investigate the effect of word frequency on words from different semantic categories. Pooling data across items with very different semantic properties − therefore increasing the variation of category-specific semantic brain activation − might have obscured effects that are specific to certain semantic categories (Pulvermüller, 1999), such as the word frequency effects for action-related and visually related words reported in this study.

The interpretation of metabolic imaging data is inherently limited by the inertia of the haemodynamic response. Importantly in the context of our study, it is generally difficult to attribute activation patterns to either early processing stages that are central to the recognition of written words or later post-access processes, which might reflect deliberate associations or mental imagery elicited in response to written words. Electrophysiological imaging studies, for example using electro- or magnetoencephalography (EEG/MEG), address this issue exploiting their millisecond temporal resolution. It is commonly argued that the earlier an effect occurs in the signal, the more likely it is to reflect elementary or automatic processes (Pulvermüller *et al*., 1996; Sereno & Rayner, 2003; Hauk & Pulvermüller, 2004b; Shtyrov *et al*., 2004; Pulvermüller, 2005; Hauk *et al*. 2006; Barber & Kutas, 2007; Kiefer *et al*., 2007). Several studies have reported category-specific effects within 250 ms after stimulus onset (Dehaene, 1995; Pulvermüller *et al*., 1995; Hauk & Pulvermüller, 2004b). Unfortunately, their comparatively low spatial resolution does not allow the inference that these early modality-specific effects arise from the same neural systems that are activated in metabolic imaging techniques. Although some correspondence between metabolic and electric brain activity has been demonstrated (Logothetis, 2002; Shmuel *et al*., 2006), and methods to constrain EEG/MEG source estimation are currently under development (Dale *et al.*, 2000; Liu *et al*., 2002), it is no simple matter to link metabolic activation spots to EEG/MEG components or source estimates. Thus, methods that verify the functional significance of activation patterns in each of these modalities separately are still required, such as the correlation with word frequency as suggested in our paper.

We argued that differential modulation of brain activation by word frequency for action-related and visually related words supports the view that these brain activations reflect aspects of lexico-semantic processing, rather than mental imagery processes. A few previous studies approached the problem of disentangling conceptual/semantic processing from mental imagery in neuroimaging using semantic priming paradigms (Wheatley *et al*., 2005; Gold *et al.*, 2006). Primes and targets were presented in rapid succession (∼250 ms), with the assumption that this would not leave enough time for the prime to evoke mental images before target presentation. Priming for semantically related word pairs was observed in several brain areas, which was interpreted as evidence that these activations reflect semantic processing, rather than mental imagery processes. Although the interpretation of these results supports our views, it is still conceivable that two consciously perceived words evoke mental images in parallel or in combination. If these interact or share part of their cognitive and neuronal processes, this could lead to decreased activation for semantically related word pairs. Thus, imagery processes might similarly explain reduced responses to paired stimuli. Furthermore, the aim of many studies is to draw conclusions about single word processing, and it would be desirable to show that category-specific activation evoked by single word presentation reflects semantic processing. In this paper, we introduced an approach that can be applied to event-related studies using single stimulus presentation, and does not impose particular constraints on the experimental design.

Our interpretation is based on the assumption that the word frequency effects observed in our study reflect central stages of word recognition related to retrieval of lexico-semantic information. In the behavioural literature, this assumption has been challenged by several authors claiming that word frequency affects only post-access decision or verification stages (e.g. Balota & Chumbley, 1984; Mccann *et al*., 1988; Paap & Johansen, 1994; McCann *et al*., 2000). For example, word frequency effects were larger in a lexical decision compared with a category verification and pronunciation task (Balota & Chumbley, 1984), suggesting that it depends on the familiarity-based decision process, rather than word identification per se. The insensitivity of naming or lexical decision times to pseudohomophones (e.g. ‘brane’) with respect to base-word frequency (‘brain’) has also been interpreted as evidence that this variable does not affect lexical access (Mccann *et al*., 1988). The resilience of word frequency effects in a dual-task paradigm, where a distractor task is assumed to interfere with early stages of word recognition processes, was presented as further evidence for a late locus of word frequency effects (McCann *et al*., 2000). These studies certainly demonstrated that effects of word frequency can be modulated by task demands. However, we did not use a lexical decision task in our study, nor any other task that required our subjects to make a decision. The argument that post-lexical processes specific to tasks that require a decision (lexical, phonological or semantic decisions) are the locus of the word frequency effects does therefore not apply in this case. Furthermore, recent behavioural studies have confirmed that effects of word frequency persist even when the task was chosen in order to minimize them, e.g. using very short exposure durations (Allen *et al*., 2005). Dual-task methodology demonstrated that word frequency exerts effects at early stages of word recognition (Cleland *et al*., 2006), supporting neurophysiological evidence cited above. These are therefore considered to be an indicator for the ease of word identification or lexical access in models of word recognition (Grainger & Jacobs, 1996). It can still be argued that in our silent reading task word frequency effects appeared at the phonological or ‘lexeme’ level, as was suggested in studies on speech production (Jescheniak & Levelt, 1994). Although we cannot rule out this interpretation for general effects of word frequency, it would not have predicted the category-specific differences for action-related and visual-related words in our study.

Neuroimaging research has consistently shown that rare words elicit stronger brain responses than words with high word frequency (Fiebach *et al*., 2002; Chee *et al*., 2003; Kuo *et al.*, 2003; Kronbichler *et al.*, 2004; Carreiras *et al*., 2006), and electrophysiological studies have demonstrated that these effects can occur early after word presentation (Sereno *et al*., 1998; Assadollahi & Pulvermüller, 2003; Hauk & Pulvermüller, 2004a; Dambacher *et al*., 2006). These studies generally explain word frequency effects on the basis of lexico-semantic processing. We therefore hypothesized that if category-specific differences in brain activation arise on a lexico-semantic level, then word frequency should correlate negatively with activation amplitudes in the corresponding brain areas. This prediction was confirmed by our data.

It should be noted that word frequency is correlated with concept familiarity, i.e. with the frequency with which subjects encounter objects or actions in real life (Morrison & Ellis, 2000). One could argue that concept familiarity should also affect imagery processes. In the context of our study, it seems implausible to us that an effect of concept familiarity on mental imagery processes can explain our results, for two major reasons. (1) Subjects were not encouraged or forced by task instructions to form mental images of the objects and actions referred to by our stimulus words. It is therefore unlikely that our subjects engaged imagery processes for concepts for which this is difficult to do. Imagery is more likely to occur for concepts for which this can be accomplished with relatively little effort, i.e. concepts with high familiarity. This would predict more brain activation for concepts with higher familiarity, i.e. a positive correlation with Frequency, which is the opposite of what we found. (2) We included measures for imageability and action-relatedness into our analysis, and effects of these variables were partialled out. This directly aims at removing any effects that are caused by mental imagery of objects or actions, independently of the underlying mechanism, as long as they are reflected by the subjects' rating. Future studies should attempt to disentangle the effects of different types of word frequencies and concept familiarity in more detail. This may require selection of ‘awkward’ stimulus items, i.e. those that often occur in written or spoken language, but almost never in real life. We hold the view that the novel procedure applied in our present study presents a way to obtain essential information about the nature of category-specific activations from neuroimaging data.

### Effects of semantic variables

In addition to providing novel results on category-specific effects of word frequency, we also aimed at corroborating previous results on semantic variables imageability and action-relatedness. Existing data on category-specific activation for highly imageable words are still inconsistent. In general, it appears that concrete and highly imageable words activate more brain areas than abstract and low imageability words, but with considerable variability (Fiebach & Friederici, 2004; Scott, 2004). Stronger activation in fusiform brain areas for imageable words has been reported by several previous studies (Wise *et al.*, 2000; Fiebach & Friederici, 2004; Sabsevitz *et al*., 2005), but some failed to find such effects (Jessen *et al.*, 2000; Binder *et al*., 2005). In our study, we found the most reliable modulations of brain activation for Imageability at almost symmetrical locations in the left and right fusiform gyri. We could also show that these were very close to activation spots that were previously reported for object processing (Chao *et al*., 1999; Martin & Chao, 2001), although slightly more anterior (∼10 mm) than the mean coordinate obtained from the Chao *et al*. (1999) study. It has been demonstrated that fusiform gyrus itself is not a homogenous structure, e.g. that the lateral–medial dimension distinguishes between objects and tools, and the posterior–anterior dimension between simple and more complex visual features (Martin, 2007). In our data, there is considerable overlap between our activated clusters and previous results reported for object-related processing. Such results, as well as the double dissociation between brain loci for action-relatedness and imageability on the one hand and for action- and visually related words on the other, demonstrates that words referring to highly imageable concepts share a neuronal substrate with the systems involved in perceiving the corresponding objects.

The most reliable positive correlations with Action-relatedness were found in two adjacent spots in the superior and middle temporal gyrus. These areas have previously been associated with naming action-related objects (Martin *et al*., 1996; Tranel *et al*., 2005), verb and action word processing (Wise *et al.*, 1991; Martin *et al*., 1995; Perani *et al.*, 1999; Devlin *et al.*, 2002; Davis *et al*., 2004), and action observation (Grezes *et al*., 2003; Aziz-Zadeh *et al*., 2006). Martin *et al*. (1996) suggested that this region may be related to knowledge about biological motion, i.e. motion of animate agents such as during tool use (Puce & Perrett, 2003). In support of this hypothesis, it has been reported that eye, mouth and hand movements activate the posterior temporal cortex (Pelphrey *et al*., 2005). This is in line with the observation that neurons in the superior temporal sulcus in monkeys show activation in action observation, but do not have motor and therefore no ‘mirror’ properties (Rizzolatti & Craighero, 2004), and rather have been associated with perception of biological motion (Perrett *et al.*, 1989; Jellema & Perrett, 2003). An fMRI study in humans showed that activation in the superior temporal sulcus can also be evoked by imagery of motion, again raising the question what level of processing such activation reflects in action-word comprehension (Grossman & Blake, 2001). In a *post hoc* analysis of their fMRI data, Davis *et al*. (2004) provided evidence that this region is modulated by action-relatedness of words in a one-back synonym monitoring task. We obtained this effect for silent single word reading, without explicit semantic task demands, and in addition showed that activity in this area for action words is modulated by word frequency.

Further activation for Action-relatedness was located in hand premotor cortex of the left hemisphere. Although relatively weak, the lateralization and approximate location of this activation indicate that it corresponds to motor areas for the dominant right hand. It must be noted here that action-relatedness, as determined from our rating study, captured general action-related aspects (i.e. related to any body part or type of movement) rather than specific ones such as manipulability. It might therefore be surprising that we found activation specifically in the hand motor cortex of the dominant hand at all. Modulation of hand motor cortex activity by action observation has been documented previously by transcranial magnetic stimulation (TMS) (Fadiga *et al*., 1995; Strafella & Paus, 2000; Aziz-Zadeh *et al*., 2004) and neuroimaging studies (Buccino *et al.*, 2001; Grezes & Decety, 2001). With respect to language, several behavioural and TMS studies have provided evidence that hand movements and hand motor areas are modulated by perception of speech in general and hand action-related words or sentences in particular (Seyal *et al*., 1999; Floel *et al*., 2003; Buccino *et al.*, 2005; Pulvermüller *et al*., 2005; Boulenger *et al.*, 2006), although the involvement of hand motor cortex in silent reading is still unclear (Seyal *et al*., 1999; Meister *et al.*, 2003). Our data add further evidence that hand motor areas play a special role for general action understanding. It is also interesting to note that hand motor cortex is located in between face and leg motor areas, and might therefore capture the overlap of activations produced by all three action word categories.^1^ It is therefore still possible that action-relatedness affects hand, face and leg motor areas, but was only detected in the hand area due to this overlap. In this case, hand motor cortex would not be ‘special’ with respect to action-relatedness. The correlation between Frequency and activation to action-related words in this area did not exceed our statistical threshold. The absence of such an effect can be explained by the fact that our action-word category comprised words referring to hand/arm, foot/leg as well as mouth/head actions. Different action word categories have been shown to activate different parts of the motor cortex in previous studies (Hauk *et al*., 2004; Tettamanti *et al.*, 2005; Aziz-Zadeh *et al*., 2006). Variability across stimuli might therefore have obscured this effect. Our data demonstrate the feasibility of our approach for areas that are most consistently activated by action- and visually related words, but do not exclude the possibility that other brain areas exhibit category-specific effects for further subcategories of words. This issue should be addressed by future investigations. Interestingly, Frequency of action words also modulated brain areas in the left inferior parietal lobule and left inferior frontal cortex, which were associated with complex actions or action recognition in previous studies (Jeannerod *et al*., 1995; Rizzolatti & Luppino, 2001). This result suggests that correlating neural activity with word frequency, separately for different word categories, has the potential to reveal brain areas that are not found in conventional analyses.

### Conclusion

Our study is the first to show that the frequency of occurrence of written words modulates brain activation for different word categories differentially. We interpret this as evidence that this activation reflects lexico-semantic processing stages. This has important implications for psychological conceptions of word meaning, as it shows that word meaning is grounded in systems that serve to interact with the external world, rather than in purely symbolic or abstract codes. Our approach of studying the effects of lexical variables on category-specific responses offers new perspectives for neuroimaging research into the neural basis of word and object processing.

## Footnotes

1 We are grateful to an anonymous reviewer for offering this explanation.

## Abbreviations

anova: analysis of variance
BOLD: blood-oxygen-level-dependent
EEG: electroencephalogram
EPI: echo planar images
fMRI: functional magnetic resonance imaging
MEG: magnetoencephalography
MNI: Montreal Neurological Institute
SOA: stimulus onset asynchrony
SVC: small volume correction
TMS: transcranial magnetic stimulation
